# The composite face effect is robust against perceptual misfit

**DOI:** 10.3758/s13414-021-02279-0

**Published:** 2021-04-22

**Authors:** David Kurbel, Bozana Meinhardt-Injac, Malte Persike, Günter Meinhardt

**Affiliations:** 1 Johannes Gutenberg University, Mainz, Germany; 2 Catholic University of Applied Sciences (KHSB), Berlin, Germany; 3 RWTH, Aachen, Germany

**Keywords:** Feature integration, Composite face effect, Congruency effect, Selective attention, Face perception

## Abstract

The composite face effect—the failure of selective attention toward a target face half—is frequently used to study mechanisms of feature integration in faces. Here we studied how this effect depends on the perceptual fit between attended and unattended halves. We used composite faces that were rated by trained observers as either a seamless fit (i.e., close to a natural and homogeneous face) or as a deliberately bad quality of fit (i.e., unnatural, strongly segregated face halves). In addition, composites created by combining face halves randomly were tested. The composite face effect was measured as the alignment × congruency interaction (Gauthier and Bukach *Cognition, 103*, 322–330 2007), but also with alternative data analysis procedures (Rossion and Boremanse *Journal of Vision, 8*, 1–13 2008). We found strong but identical composite effects in all fit conditions. Fit quality neither increased the composite face effect nor was it attenuated by bad or random fit quality. The implications for a Gestalt account of holistic face processing are discussed.

## Introduction

When observers have reached high levels of expertise with individual members of an object category, they often experience difficulty in judging object parts independently (Gauthier, Curran, Curby, & Collins, 2003). Particularly, this is true for human faces (Tanaka & Farah, 1993; Tanaka & Sengco, 1997). The strong contextual influence may reflect “holistic” processing—the tendency to process faces as indivisible wholes (Rossion & Boremanse, 2008; Rossion, 2013). Joint processing of face parts makes face processing highly efficient in tasks requiring face identity recognition (Wang, Li, Fang, Tian, & Liu, 2012; DeGutis, Wilmer, Mercado, & Cohan, 2013), or discrimination (Ellis, Shepherd, & Davies, 1979; Meinhardt-Injac, 2013). However, it is disadvantageous when just some face parts have to be processed while ignoring others, since the perceptual appearance of facial features contingently changes with the embedding facial context. A prominent example is the “presidential illusion” (Sinha & Poggio, 1996), which shows that it is difficult to identify that the inner face parts belong to Bill Clinton if the outer face parts (ears, hair, and face outline) come from Al Gore. The failure of selective attention to parts thus offers methodological access to the principles of feature integration in face perception (Maurer, Le Grand, & Mondloch, 2002; Richler & Gauthier, 2014; Rossion, 2013).

The most popular experimental paradigm used to study the failure of selective attention to face parts is the composite face paradigm (Young, Hellawell, & Hay, 1987), which has been refined over the past three decades. In the composite task, faces are composed of top and bottom halves, which may stem from different face identities. Two such composite faces are presented and observers have to evaluate the identity of either top or bottom halves. When two identical top halves are combined with different bottom halves, the top halves appear different (Rossion, 2013, p. 2). Because the two faces are indeed different as integrated wholes, the misperception of part identity may be due to an inherent tendency of observers to evaluate faces as unified wholes rather than as an assembly of parts (Rossion & Boremanse, 2008). Spatial misalignment of face halves is one way to disrupt holistic integration, thus enabling observers to judge the identity of the attended face halves free of the influence of the non-attended halves (Schiltz & Rossion, 2006). Although there are different theoretical accounts of the composite face effect, there is agreement that the performance difference obtained between aligned and non-aligned conditions is crucial for judging the failure of selectively attending the target face half (Rossion 2013, p. 4; McKone et al., 2013; see Discussion in Richler et al.,^2014^).

Misalignment disrupts the configural order of face elements—the spatial arrangement of top and bottom parts does not fit the typical face template anymore. The natural “Gestalt” of a face is broken, which may preclude integration of face parts (Rossion, 2013, pp. 32). As expected from a Gestalt account, it was found that moderate degrees of misalignment caused a strong decline in the composite face effect (Languesse & Rossion, 2011). Zhao, Bülthoff, and Bülthoff (2016a) measured the composite effect for faces and non-face line drawings, which realized the Gestalt principle of good continuation across top and bottom halves. Results revealed a composite effect for the non-face line-drawings, which was as strong as the composite effect obtained for faces. In a control experiment, the authors reduced the Gestalt information by using dot patterns that were harder to group. In line with the prediction from Gestalt-based grouping of top and bottom object parts, a smaller composite effect resulted when good continuation and connectedness of line structures across both object halves were attenuated. These results let authors surmise that Gestalt-based perceptual grouping may also enter in face processing. Acknowledging the role of expertise, the authors proposed a dual-route account of holistic face processing, assuming that experience-based knowledge defines what constitutes a facial Gestalt (top-down route), while Gestalt laws of grouping (i.e., similarity, good continuation, and connectedness) guide local feature integration (bottom-up route, see Zhao et al., 2016a, pp. 220).

Support for this account came from recent experiments of Curby and colleagues. Curby, Goldstein, and Blacker (2013) showed that the composite face effect is attenuated when background frames drawn around top and bottom face halves are misaligned and disagree in color, suggesting that task-irrelevant grouping cues affect holistic integration. To study whether holistic processing of faces and non-face Gestalt objects interact, (Curby & Moerel, 2019) overlaid faces with the non-face Gestalt-line drawings used by Zhao et al., (2016a). When misaligned line drawings were used as the background stimuli, the composite face effect was enhanced, while it was reduced when the background line drawings were aligned. Using faces as the background stimuli and measuring the composite effect for non-face objects yielded similar results. These findings indicate a common holistic mechanism for faces and non-face objects at an earlier, perceptual level of stimulus processing.

A Gestalt account of face perception would predict that holistic integration depends on the perceptual fit of face halves. If top and bottom halves have highly similar skin tone and texture, and if shapes of nose and face outline smoothly continue from top to bottom halve without noticeable transition at the boundary line, holistic integration should be strong. Consequently, a strong composite effect is expected for a good fit of face halves. On the other hand, if face halves do not integrate well with local shape breaks, dissimilar skin tone and texture, the salient boundary between the halves amplifies their separation, which should diminish, or even preclude, holistic integration. Hence, an attenuated composite effect is expected for badly fitting halves. Anticipating relevance of good continuation and connectedness in all facial aspects, some authors select faces such that upper and lower halves fit well (McKone et al., 2013; Rossion, 2013), and take action to avoid any noticeable transition at the mid border (Rossion and Retter, 2015).

One may, however, challenge the claim that holistic integration of face parts depends on good perceptual join quality. Reviewing findings about the functional specificity of face-tuned brain regions shows that the fusiform face area (FFA), which has preference for upright faces (Yovel & Kanwisher, 2004), also responds with orientation selectivity to crude face-like patterns, such as cartoons and two-tone “Mooney faces” with highly variable low-level image properties (Tong et al., 2000; see review by Kanwisher & Yovel, 2006). Face-like processing for rather crude face stimuli was also shown in binocular rivalry (Stein, Peelen, & Sterzer, 2011). From these results one might expect just the opposite of what the Gestalt account suggests. If face-specific processing is invoked even by coarsely face-like stimuli, the composite face effect might not, or just modestly, depend on perceptual fit in composite faces, tolerating segregation borders among face halves with clear breaks in connectedness and good continuation from top to bottom. Hence, the question arises whether the visual system integrates face halves into a unique facial representation *even though* the halves have dissimilar local shape attributes, skin tone, and texture, indicating that they apparently stem from different original faces.

In the present study, we investigated whether the perceptual fit of halves affects the composite face effect. Since the misaligned control condition is used in two varieties in current studies on the composite face effect (both first and second composite image misaligned versus first composite image aligned and second composite image misaligned) we tested with both varieties. Further, we used the complete design of the composite face paradigm (Gauthier & Bukach, 2007), and applied several analysis methods to ensure that our findings covered the relevant composite effect measures currently in use.

## General methods

### Stimuli and stimulus matching procedure

Two hundred greyscale face images from the Tübingen MPI Face Database (Troje & Bülthoff, 1996) were first cut into top and bottom halves of constant height. Stimulus construction then set out to reassemble a subset of those halves into composites for the main experiments. We aimed for a set of 48 composites, 16 were supposed to exhibit a seamless fit between the top and bottom half, 16 were intended to have a deliberately bad fit, and the last 16 had random fit between halves. Composite faces were formed just by selection, no image manipulation or contrast normalization techniques were applied.

To find half pairings representing good and bad fit quality, we used a computer-assisted match-making procedure. One of the authors (MP) and a research assistant completed the procedure as observers. The software first randomly selected a top half, and combined it with a randomly selected bottom half. The two observers, unaware of each other’s evaluations, judged the bottom half either as a near-seamless fit for the top half or not. If at least one of the observers rejected the combination as a near-seamless fit, the bottom half was discarded and the next bottom half was selected at random. Only if both observers deemed the fit near-seamless was the bottom half marked as a match for the top half. When four matching bottom halves were found, a new top half was selected at random and the procedure was started anew. Before sampling bottom halves at random, the bottom halves that fit to the previous top half were tried. This was done to ensure that there were bottom halves that fitted to at least two top halves, as it is required to maintain the logic of the complete design (see Fig. 3). After 16 top halves with fitting bottom half subsets were created, it was checked that for any top half there was at least one other top half that shared at least one same bottom half in their subset of fitting bottom halves. The same procedure was applied to create bad fitting composites. Face halves from female and male models were never mixed into a composite face. In the experiment, image halves were always taken from one specific pair out of the preselected set of matching pairs, and then a set of four trials (same-congruent, same-incongruent, different-congruent, different-incongruent; see section below) was created for each selection.

**Fig. 1:**
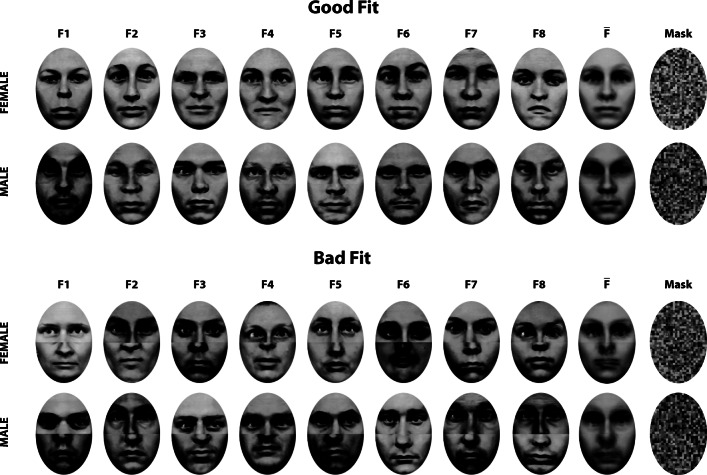
The composite face sets representing good and bad fit of halves. Sixteen face instances were selected, eight male and eight female. The last but one column shows the average faces (centroids) of each row, the last column shows examples of randomly ordered 5 × 5 pixel blocks sampled from the average faces, which were used as masks

For creating masks, average faces (set “centroids”) were calculated by taking the mean grey values from all composite faces of each fit quality face set, but separately for each gender. Scrambled face masks were created by randomly assembling 5 × 5 pixel blocks sampled from the corresponding centroid. Only true face parts within the elliptical frame were used. This was done anew whenever a mask was presented in a trial. Face sets, centroids, and masks for good and bad fit conditions are shown in Fig. 1.

### Face image statistics

Albeit low-level image statistics can only capture some basic aspects of facial integrity, they may give important clues to the tolerance of holistic face processing across basic face image properties in the context of fit quality. We report contrast and homogeneity measures to characterize and to compare the face sets for good and bad fit.

#### Contrast measures

As recommended for complex images, we calculated RMS contrast, *C*_*RMS*_, defined as the standard deviation of normalized grey values, $\hat {u}=u/255$, $0\leq \hat {u}_{i} \leq 1$ (Peli, 1990). Since abrupt luminance transitions at the face halve borders may affect observers’ rating of fit quality, we measured the average luminance of the last three pixel rows of the top half and of the first three pixel rows of the bottom half and then calculated Michelson contrast, *C*_*M*_ = (*l*_*top*_ − *l*_*bot*_)/(*l*_*top*_ + *l*_*bot*_), to describe the border luminance step. The luminance of the pixel grey values was obtained from a calibration measurement for the actual contrast and brightness settings of the monitor (see Apparatus).

#### Homogeneity measures

The variance of faces within each set was described by measuring the deviation of grey values of each face *F*_*ij*_ from the set centroid $\overline {F}_{j}$ (see Fig. 1), calculated by taking the square root of the second central moment, $D_{ij}=\sqrt {\frac {1}{N}{\sum }_{x,y}\left (F_{ij}(x,y)-\overline {F}_{j}(x,y)\right )^{2}}$. Further, we calculated the cross-correlation matrix for each fit quality set, separately for male and female gender (i.e., for each row of faces shown in Fig. 1). Then, the average cross-correlation of each composite face with the remainder faces of the set, $\overline {r}_{i^{\prime }i}$, was computed, taking Fisher *Z*- transforms of the Pearson product-moment correlations before averaging and then calculating the back-transform. Also, the significance test for the average correlations was executed with Fisher *Z*-transforms of the average correlation measures.

Table 1 shows descriptive statistics and *t* test results for comparison across fit quality. The data show that good and bad fitting face composites almost coincided in the global RMS contrast measure. However, they differed significantly in local contrast at the face half border. Means and confidence intervals for *C*_*M*_ indicate almost seamless face half transitions in the “Good Fit” set, while composites from the “Bad Fit” set showed salient luminance steps at the transition of halves. A Cohen’s *d* of 1.39 indicates that the sets differed strongly in this respect.

**Table 1:** Descriptive statistics of contrast measures and homogeneity measures and *t* test results for comparison across fit quality

Measure	Fit quality	*μ*	*s*_*e*_	*CI*_95*%*_	Δ*μ*	*t*(30)	*P*	*d*
*C*_*RMS*_	bad	0.191	0.003	[0.184 , 0.198 ]	0.002	0.49	0.631	0.17
	good	0.189	0.003	[0.182 , 0.195 ]				
*C*_*M*_	bad	0.138	0.021	[0.094 , 0.182 ]	0.119	3.92	< 0.001	1.39
	good	0.019	0.021	[-0.025 , 0.062 ]				
*D*_*ij*_	bad	33.31	1.216	[30.82 , 35.79]	6.642	3.86	< 0.001	1.37
	good	26.66	1.216	[24.18 , 29.15]				
$\overline {r}_{ii^{\prime }}$	bad	0.521	0.014	[0.492 , 0.549 ]	-0.177	-9.72	< 0.001	3.44
	good	0.698	0.014	[0.669 , 0.727 ]				

The table shows mean (*μ*), standard error (*s*_*e*_), 95*%* confidence interval (*CI*_95*%*_), mean difference (Δ*μ*), *t*-statistic with degrees of freedom (*t*(*df*)), significance level (*P*), and Cohen’s effect size index (*d*), for RMS contrast (*C*_*RMS*_), Michelson contrast at the face half border (*C*_*M*_), deviation from class centroid (*D*_*ij*_), and average cross correlation of faces $(\overline {r}_{ii^{\prime }})$

The homogeneity measures reveal that the set of composite faces with good fit was much more homogeneous than the set of badly fitting composites. The latter deviated stronger from their set centroid, and also their average cross-correlation was significantly lower compared to good fitting composites. An average cross-correlation of 0.698 revealed a high degree of coherence within the “Good Fit” set, indicating that the spatial grey value distribution of one composite face is predicted from another one with 49*%* explained variance. In contrast, just 27*%* of explained variance was reached for badly fitting composites. A Cohen’s *d* of 3.44 reflects a very strong difference of good and badly fitting composites in the average cross-correlation measure. Since this measures the correspondence of grey values at the same points within the face, it is sensitive to deviations in the spatial distribution of hue and skin texture of two faces. Relative position and size of cardinal features (eyes, nose, mouth) will also affect the cross-correlation measure, since grey values change significantly in these regions. Thus, a high average cross-correlation indicates that a face set is quite homogeneous both in the spatial distribution of texture and hue, but also in size and relative positioning of cardinal features.

### Measure of the composite effect

**Fig. 2:**
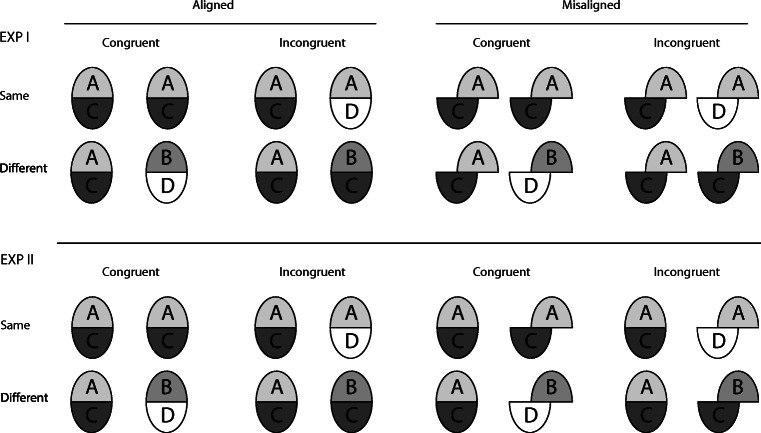
Overview of the complete design, and illustration of the two varieties of the misaligned control condition realized in Experiment 1 (*upper panel*) and 2 (*lower panel*)

To test effects of fit quality requires a valid measure for the composite effect. In the two experiments reported here, we used a comprehensive experimental design, introduced by Gauthier and Bukach (2007) as the “complete design” (CD). It is depicted in Fig. 2. The major characteristic of the CD is that it uses “congruent” and “incongruent” trials, which are orthogonally balanced with “same” and “different” trials (see Fig. 2). In congruent trials, the unattended halves agree when the attended ones agree, and they disagree, when the attended halves disagree, which means that the identity relation of target and non-target parts is the same. As a result, the decisional outcome is the same if the observer relies on identity/nonidentity of only the target halves, or of the integrated whole faces. In incongruent trials, on the other hand, the unattended halves disagree when the attended ones agree, and vice versa, which means that the identity relation of target and non-target parts is orthogonal. As a result, the observer’s decision will be at chance level if she/he relies on identity/nonidentity of the integrated whole faces instead of only the target halves, since the wholes are *always* different in incongruent trials (see Fig. 2, see also Meinhardt et al., 2014).

Hence, for a “holistic” observer, who has difficulty to selectively attend only the target half but is prone to evaluating the properties of the integrated whole face instead, different effects are expected for congruent and incongruent face half pairings. These can be assessed when face halves are presented aligned and also misaligned.

Compared to misaligned presentation where perceptual fusion of halves is precluded, the holistic observer can rely on cues from the whole face in aligned presentation to decide whether stimuli are same or different. Since there is full agreement/disagreement in congruent trials, accuracy should be higher in aligned compared to misaligned presentation. In incongruent trials, however, using cues from the non-target halves interferes with a correct decision about the identity of the target halves, which leads to more errors compared to misaligned presentation. Hence, by manipulating the congruency relation two distinct effects of alignment are captured. First, a performance benefit for aligned compared to misaligned presentation, which is expected for congruent trials. Second, a performance decline for aligned compared to misaligned, expected for incongruent trials. Both effects are contained in the alignment × congruency interaction. Plotting performance means for congruent and incongruent trials, having the two alignment conditions on the abscissa, must result in a scissor-like pattern, indicating a disordinal type of interaction which reflects the two opponent effects of alignment for congruent and incongruent trials. Typical results are shown, for example, in Zhao et al., (2016a), and are also found in the Results part of this study.

**Fig. 3:**
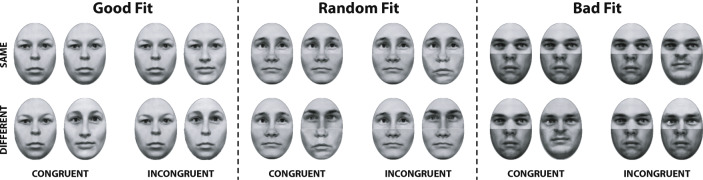
Composite face examples corresponding to the trial assignments of the complete design shown in Fig. 2 for good, random, and bad fit of halves in composite faces

Measuring the composite effect by the alignment × congruency interaction yields a robust and redundant measure, since both better performance in congruent trials and the increase of errors in incongruent trials are implied by an integrated processing of face halves. Hence, from a Gestalt account of holistic integration, a stronger alignment × congruency interaction for a good fit, compared to a random or bad fit of halves is expected (see stimulus examples in Fig. 3). In ANOVA, such a modulatory effect of fit quality would be reflected by a significant alignment × congruency × fit quality interaction.

### Alternative measures

Some authors used only a subset of trials contained in the complete design, namely “same” trials only in the incongruent variety (agreement of target half, disagreement of non-target half), and “different” trials only in congruent identity relation (disagreement of both target and non-target halves; Goffaux and Rossion, 2006; Rossion & Boremanse, 2008). The major reason for doing so is that authors favored a specific concept of the composite face effect, assuming that it manifests in a perceptual “illusion”, which lets the same top halves appear different when they are paired with different bottom halves, while different top halves are seemingly not perceived as more identical when associated with identical bottom parts (Rossion & Boremanse, 2008; Rossion, 2013). In this approach, the composite face effect is reflected by the performance difference between aligned and misaligned presentation in incongruent-same trials. In the present study, we report results also for this approach, being aware of reservations that should be taken into account when interpreting the results.^1^ Since, in this account of the composite effect, the alignment effect for incongruent-same trials is used as the crucial measure, a modulatory effect of fit quality would be reflected by a significant alignment × fit interaction.

### Experimental varieties of the misaligned control condition

For the composite paradigm, two different varieties of the misaligned control condition are currently in use. In the majority of studies, the misaligned control condition is realized by presenting both the first and the second composite face image of a trial misaligned (see Rossion, 2013). However, in several other studies, the first composite face image (“study face”) was aligned and the second (“test face”) misaligned, among them the studies of Zhao and colleagues that reported the Gestalt effect for non-face composites (Richler, Gauthier, Wenger, & Palmeri, 2008; Richler, Cheung, & Gauthier, 2011; Richler, Floyd, & Gauthier, 2014; Zhao et al., 2016a; Zhao, Bülthoff, & Bülthoff, 2016b).^2^ Since this difference may affect face processing in a trial (see Richler et al., 2008), and since we wanted to ascertain that potential effects of fit quality on the composite face effect are not confounded with the variety of the misaligned control condition, we realized both varieties. In Experiment 1, we used misalignment for study and test, while only the test face was misaligned in Experiment 2 (see Fig. 2). In both experiments, the same stimulus material was used and the same experimental procedures were applied. Different subjects participated in either experiment.

### Apparatus

Stimuli were generated with Matlab R2014a and displayed on an Eizo ColorEdge CG 2420 color monitor. Face stimuli had a pixel size of 127 × 186 pixels, which corresponded to about 3.5^∘^× 4.8^∘^ of visual angle. The mean luminance of the screen was 91.5 cd/m^2^, and screen Michelson contrast was 0.981. The refresh rate of the monitor was 60 Hz and the pixel resolution was set to 1920 × 1200 pixels. No gamma correction was used, but the monitor response function was measured. To do so, lookup-table entries (*u*) were loaded with equal settings for R, G, and B and a central square field of 300 × 300 pixels was successively set to each of 16 selected grey values taken from the range 0–255 while the luminance (*l*) of the field was measured (LMT1003 photometer, Lichtmesstechnik Berlin GmbH). The resulting function was fitted by the second-order polynomial *l*(*u*) = 1.4 + 0.312203*u* + 0.00306018*u*^2^ with good accuracy (*R*^2^ = 0.991). The function was used for calculating Michelson contrasts (see above). The room was darkened so that the ambient illumination approximately matched the illumination of the screen. Stimuli were viewed binocularly at a distance which varied in the range of 60 to 70 cm since no chin rest was used. Participants gave responses with their dominant hand via an external keypad.

### Experimental design

Both Experiment 1 and Experiment 2 followed a 3 (Fit; good, bad, or random) ×2 (Congruency; congruent or incongruent) × 2 (Alignment; aligned or misaligned) design. Trials with good, bad, or random fit quality formed separate experimental blocks. With 16 same and 16 different trials, an experimental block comprised 128 trials. To go through a block took about 10 min. The sequence of the three fit quality blocks was counterbalanced across subjects.

### Procedure

A temporal same/different forced choice matching task was used. The task of the observers was to judge the identity of the top halves of two successively presented faces. The observers were informed that face pairs could differ in the top or the bottom halves, or in both, and that the identity of just the top halves had to be judged. The temporal sequence of events in a trial was: fixation mark (750 ms) - blank (300 ms) - study image (800 ms) - mask (400 ms) - test image (250 ms) - mask (400 ms) - blank frame until response. To preclude pixel matching, study and test images were independently displaced in both horizontal and vertical directions by a random jitter of ± [0,50] pixels. In trials with misaligned image halves, the halves were displaced by 0.5 × image width about the center, in the same direction for study and test image. The shift direction (leftward or rightward) varied randomly across trials.

**Fig. 4:**
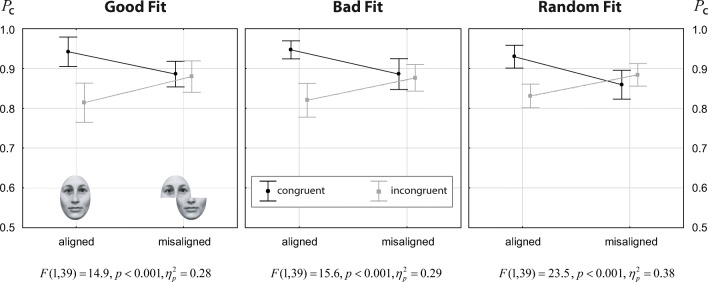
Results from Experiment 1: response accuracy (proportion of correct responses) for faces as a function of alignment for congruent (*black symbols*) and incongruent (*gray symbols*) trials. *Error bars* indicate 95*%* confidence intervals of the means. At the panel bottoms, the results for the alignment × congruency interaction for each individual fit condition are displayed, obtained by analyzing only the given fit condition with ANOVA

Richler and colleagues controlled presentation times by masking the test image with several delays up to 800 ms, and found that the composite effect was maximal (Richler, Mack, Gauthier, & Palmeri, 2009) and face-specific (see Fig. 5 in Richler et al., 2011) for presentation times of the test face of 183 ms and beyond. Using the same paradigm and varying attentional task demands, Meinhardt-Injac, Persike, and Meinhardt (2014) confirmed a large composite effect for test face timings of 50 ms and 233 ms while the effect declined for more relaxed presentation times (633 ms) when attentional task demands were low, as in this study (see ibid., Fig. 4, lower left panel). Results obtained with a context congruency paradigm (Meinhardt-Injac, Persike, & Meinhardt, 2010; Meinhardt-Injac, Persike, & Meinhardt, 2011)confirm this finding, and suggest that observers are able to exploit additional temporal resources to increase performance in the incongruent condition, which indicates better attentional control and less holistic integration. Since here we addressed potential decline of the composite effect as a result of poor fit quality, it was important to grant optimal conditions for obtaining a maximal composite effect by selecting a shorter presentation time for the test face and precluding potential postprocessing from iconic memory with effective masking.

### Ethics statement

Participants were recruited through in-house messaging boards. Prior to the experiment, participants were informed about the course and expected duration of the experiment. They received a general description of the purpose of the experiment but not about specific outcome expectations. All participants signed a written consent form according to the World Medical Association Helsinki Declaration and were informed that they could withdraw from the experiment at any time without penalty. Noninvasive experimental studies without deception do not require a formal review by the institutional ethics committee provided the experiment complies with the relevant regulations and legislation which was carefully ascertained by the authors. After completing the experiment, a summary of their individual data was shown to the observers and the results pattern explained.

## Experiment 1: Composite effect with misaligned control in study and test

### Participants

The experiment was completed by *n* = 44 participants, 28 of them were female. The age range was 19–35 years. Participants received course credits or were paid upon completing the experiments (5 euros). All observers had normal or corrected-to-normal vision.

### Dependent measures and data clearing

The accuracy of judgements was measured by the proportion of correct responses taken from both correct same and correct different trials. Out of the 44 participants, 40 were included in the data analysis. Four subjects were dropped because they showed near chance performance of below 60% correct in the easiest conditions where near-perfect performance is attained by most observers (i.e., aligned-congruent).

### Results of Experiment 1

Figure 4 shows the data for the three fit conditions as alignment × congruency interaction plots. The corresponding ANOVA results are summarized in Table 2. There were only two significant effects: a strong main effect of congruency, and a strong alignment × congruency interaction. The quality of fit had no main effect, and did not modulate the congruency effect, nor the alignment × congruency interaction. This general pattern becomes obvious in Fig. 4. The plots are highly similar for the three fit conditions, showing just marginal differences. Testing the alignment × congruency interaction for each fit quality condition individually (see captions of Fig. 4) showed ${\eta _{p}^{2}}$ values in the range of 0.28 to 0.38. These results comply with the effects sizes reported from a recent meta-analysis of 48 studies using the complete design, where an average effect size of ${\eta _{p}^{2}}=0.32$ for the alignment × congruency effect was found (Richler & Gauthier, 2014). We further explored the alignment × congruency effect by testing the congruency effect separately for aligned and nonaligned trials. Results showed a strong congruency effect for aligned (*t*(39) = 8.45,*p* < 0.001,*d* = 1.34) and no congruency effect for misaligned trials (*t*(39) = 0.29,*p* = 0.775,*d* = 0.05).

**Table 2:** ANOVA results for testing the effects of fit, congruency and alignment in Experiment 1

Source of variation	SS	df	$\hat {\sigma }^{2}$	F	P	$\hat {\eta }_{p}^{2}$
Alignment (A)	0.001	1	0.001	0.05	0.827	0.001
Error	0.419	39	0.011			
Congruency (B)	0.394	1	0.394	41.18	< 0.001	0.514
Error	0.373	39	0.010			
FIT (C)	0.003	2	0.002	0.09	0.917	0.002
Error	1.469	78	0.019			
Alignment × Congruency	0.438	1	0.438	47.78	< 0.001	0.551
Error	0.358	39	0.009			
Alignment × Fit	0.004	2	0.002	0.30	0.743	0.008
Error	0.462	78	0.006			
Congruency × Fit	0.025	2	0.013	1.36	0.263	0.034
Error	0.729	78	0.009			
A × B × C	0.000	2	0.000	0.01	0.987	0.000
Error	0.628	78	0.008			

The table shows source of variation, sum of squares (*SS*), degrees of freedom (*df* ), variance estimate ($\hat {\sigma }^{2}$), *F*- ratio, (*F*), significance level (*P*), and estimate of partial eta-squared ($\hat {\eta }^{2}_{p}$)

## Experiment 2: Composite effect with misaligned control only in test

### Participants

The experiment was completed by *n* = 49 participants, 31 of which were female. The age range was 19–28 years. Participants received course credits or were paid upon completing the experiments (5 euros). All observers had normal or corrected-to-normal vision.

### Dependent measures and data clearing

As in Experiment 1, the proportion of correct responses was measured, taken from both correct same and correct different trials. Out of the 49 participants, 44 were included in the data analysis. Five subjects were dropped because they showed near chance performance of below 60*%* correct in aligned-congruent trials.

### Results of Experiment 2

**Fig. 5:**
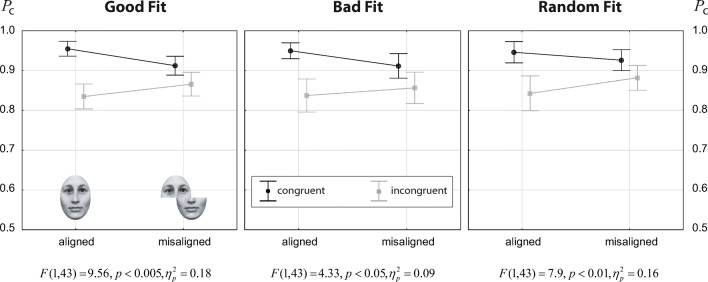
Results from Experiment 2. Conventions as in Fig. 4

Figure 5 shows the data for the three fit conditions as alignment × congruency interaction plots. ANOVA results are summarized in Table 3. As in Experiment 1, there were only two significant effects: a strong main effect of congruency, and a strong alignment × congruency interaction. Again, the alignment × congruency interaction plots were highly similar for the three fit conditions. Fit quality had no main effect, and it did not modulate the congruency effect, nor the alignment × congruency interaction. However, testing the alignment × congruency interaction for each fit quality condition individually (see captions of Fig. 5) showed ${\eta _{p}^{2}}$ values in the range of 0.09 to 0.18, indicating smaller effect size than reported in the meta-analysis of Richler and Gauthier (2014). Further exploring the alignment × congruency effect showed a strong congruency effect for aligned (*t*(43) = 11.24,*p* < 0.001,*d* = 1.69), and also a congruency effect of medium effect size for misaligned trials (*t*(43) = 4.09,*p* < 0.001,*d* = 0.61).

**Table 3:** ANOVA results for testing the effects of fit, congruency, and alignment in Experiment 2

Source of variation	SS	df	$\hat {\sigma }^{2}$	F	P	$\hat {\eta }_{p}^{2}$
Alignment (A)	0.000	1	0.000	0.09	0.771	0.002
Error	0.236	43	0.005			
Congruency (B)	0.849	1	0.849	98.77	< 0.001	0.697
Error	0.370	43	0.009			
FIT (C)	0.010	2	0.005	0.46	0.635	0.011
Error	0.938	86	0.011			
Alignment × Congruency	0.132	1	0.132	18.27	< 0.001	0.298
Error	0.311	43	0.007			
Alignment × Fit	0.009	2	0.005	0.85	0.432	0.019
Error	0.458	86	0.005			
Congruency × Fit	0.003	2	0.001	0.22	0.801	0.005
Error	0.535	86	0.006			
A × B × C	0.002	2	0.001	0.14	0.868	0.003
Error	0.522	86	0.006			

The table shows source of variation, sum of squares (*SS*), degrees of freedom (*df* ), variance estimate ($\hat {\sigma }^{2}$), *F*- ratio, (*F*), significance level (*P*), and estimate of partial eta-squared ($\hat {\eta }^{2}_{p}$)

## Comparison of Experiment 1 and Experiment 2

To reveal differential effects of the different varieties of alignment condition used in Experiment 1 and Experiment 2 we entered the data into an omnibus ANOVA with experiment (1 or 2) as a grouping factor. Results showed no main effect of experiment (*F*(1,82) = 1.12,*p* = 0.293). Besides the expected effects of congruency ($F(1,82)=131.2,p<0.001,{\eta _{p}^{2}}=0.62$) and alignment × congruency ($F(1,82)=65.3,p<0.001,{\eta _{p}^{2}}=0.44$) there was a significant alignment × congruency × experiment effect ($F(1,82)=6.39,p<0.02,{\eta _{p}^{2}}=0.07$), indicating a weaker alignment × congruency effect in Experiment 2 compared to Experiment 1. Additionally, there was a marginally significant congruency × experiment effect ($F(1,82)=3.65,p=0.059,{\eta _{p}^{2}}=0.04$), which indicated a marginally weaker congruency effect in Experiment 1 compared to Experiment 2. This was reflected by the finding that there was a congruency effect in aligned and misaligned trials in Experiment 2, while the congruency effect was limited to aligned trials in Experiment 1 (see above). No other effects were significant or marginally significant. Particularly, there were no interactions involving fit quality and/or experiment.

## Alternatives measure of the composite effect

**Fig. 6:**
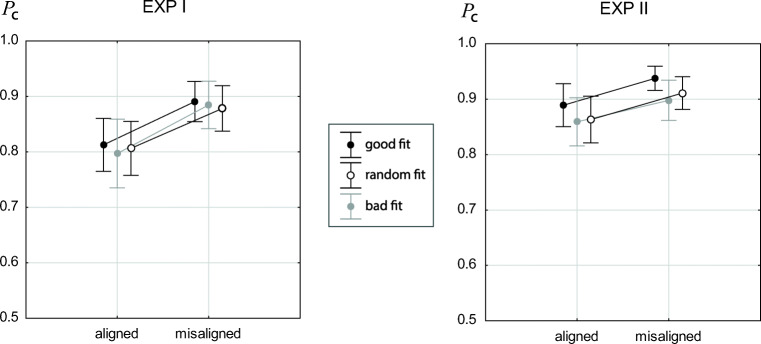
Proportion correct rates for incongruent-same trials from Experiment 1 (*left*) and 2 (*right*). *Error bars* indicate 95*%* confidence intervals of the means

Several authors use the alignment effect for only incongruent-same trials as an alternative measure of the composite effect (see Methods). We analyzed this subset of trials for potential effects of alignment and fit quality. Figure 6 shows the results as alignment × fit quality interaction plots. In Experiment 1 there was an alignment effect ($F(1,39)=21.69,p<0.001,{\eta _{p}^{2}}=0.36$), while there was no effect of fit quality (*F*(2,78) = 0.11,*p* = 0.89) and also no alignment × fit quality interaction (*F*(2,78) = 0.10,*p* = 0.90). Results for Experiment 2 aligned with results of Experiment 1 (alignment: $F(1,43)=7.85,p<0.01,{\eta _{p}^{2}}=0.15$; fit quality: *F*(2,86) = 2.34,*p* = 0.11; alignment × fit quality: *F*(2,86) = 0.12,*p* = 0.88). An omnibus ANOVA including experiment as a grouping factor revealed an effect of experiment ($F(1,82) = 8.28,p\!<\!0.001,{\eta _{p}^{2}}=0.09$), indicating better performance in Experiment 2 compared to Experiment 1. Moreover, there was a strong alignment effect ($F(1,82)=28.22,p<0.001,{\eta _{p}^{2}}=0.26$), while the alignment × experiment interaction did not reach significance (*F*(1,82) = 2.14,*p* = 0.147), indicating that the alignment effect was statistically not distinguished between the two experiments. All other effects, particularly interactions involving fit quality and/or experiment, were not significant or marginally significant. Since a main effect of experiment was obtained for incongruent-same trials, but not for all trials (see above), better performance for incongruent-same trials in Experiment 2 could result from a stronger bias towards the “same” response. To test this conjecture, we analyzed incongruent-same and incongruent-different trials (see grey symbols in Figs. 4 and 5) with omnibus ANOVA. Results showed no main effect of experiment (*F*(1,82) = 0.02,*p* = 0.889), confirming that better performance in Experiment 2 in incongruent-same trials was due to a stronger “same” bias in Experiment 2 at the costs of accuracy in “different” trials. The only significant effect of this analysis was the alignment effect ($F(1,82)=2.63,p<0.001,{\eta _{p}^{2}}=0.23$). As found for incongruent-same trials, the alignment × experiment interaction failed to reach at least marginal significance (*F*(1,82) = 2.63,*p* = 0.109), which means that flatter alignment slopes of incongruent trials in Experiment 2 compared to Experiment 1 could not be confirmed at any significance level with the given sample size, neither with incongruent-same trials (see Fig. 6), nor with all incongruent trials (see Figs. 4 and 5).

## Discussion

Studying the composite face effect for good, random, and bad fit of halves with two different varieties of the misaligned control condition has shown two major results. First, we found that fit quality had no impact on the composite face effect, measuring it as the alignment × congruency effect as well as the alignment effect for incongruent-same trials. Second, we found that the way the misaligned control condition is implemented indeed matters for the composite face effect.

### Impact of the misaligned control condition

When both study and test were misaligned (Experiment 1), a stronger alignment × congruency effect resulted, compared to presenting the study face aligned and the test face misaligned (Experiment 2). Further, the congruency effect was specific for aligned trials in Experiment 1, while congruency effects were found for both aligned and misaligned trials in Experiment 2. These differential effects were independent of fit quality, and could be revealed using the measures of the complete design. Analyzing only incongruent trials, or only incongruent-same trials, failed to show significance of the alignment × experiment interaction. This demonstrates that the complete design measure is more sensitive to changes of the composite face effect. Its stronger power is due to the fact that two opponent alignment effects (see General Methods) combine into one measure (alignment × congruency effect), while the partial design measure (alignment effect for incongruent-same trials) relies on just a single slope effect (Richler & Gauthier, 2014). Higher sensitivity to the variety of the misaligned control condition thus validates a two-component account of the composite face effect, comprising better performance in congruent and more errors in incongruent trials for aligned face composites (Cheung, Richler, Palmeri, & Gauthier, 2008). Likewise, Ventura and colleagues (Ventura et al., 2019) could reveal sensitivity of the composite face effect to global priming only when the complete design measure, but not when the partial design measure was used.

Richler et al., (2008) measured congruency effects for all combinations of alignment and misaligned in study and test with the aim to test predictions from a perceptual and a decisional account of the composite face effect. According to a decisional account, faces are encoded as a bundle of independent components, but decisions about part identity or nonidentity cannot be made independently (Wenger and Ingvalson, 2002; 2003). Since, in this account, face manipulations at test but not at study are crucial, one would expect same congruency effects when both test and study face are misaligned, compared to having study face aligned and test face misaligned (see Richler et al., 2008, right panel of Fig. 2). With respect to our study, this means that same alignment × congruency interactions are expected for Experiment 1 and Experiment 2. From a perceptual account, however, holistic processing enters both at encoding and when the encoded face is retrieved and compared to the test face representation. Therefore, we would expect a negligible congruency effect when both study and test face are misaligned, but a larger one when the study face is aligned, leading to a stronger alignment × congruency interaction in the first case (see illustration of predictions in Richler et al., 2008, left panel of Fig. 2).^3^ Our results are fully in line with the prediction from a perceptual account and contradict predictions from a decisional account, which ignores contextual dependency of face parts at the encoding stage. This result imposes important constrains for discussing the lacking effect of fit quality, since it suggests that the composite face effect relies on face processing at perceptual and encoding stages (Jacques and Rossion, 2009).

### “Gestalt” processing of faces

We studied whether the composite face effect critically hinges on the perceptual fit quality of the two combined faces halves by manipulating good continuation, connectedness and similarity of local shape, skin tone, and texture. Analyzing the data with different analysis methods used in the literature showed no effects of fit quality on the composite face effect, albeit strong segregation cues at the join of the image halves indicated that two quite distinct faces were combined in the composite stimuli. The strong and equal composite effects obtained for all fit quality conditions indicate that the perceptual fit of halves has no impact on the contextual interaction of face parts.

The complete absence of any modulatory effects of fit quality in the behavioral test of holistic processing is, indeed, stunning. At first glance, this finding poses problems for accounts assuming a pivotal role of Gestalt rules for building a unique facial representation from two face halves (Rossion, 2013; Zhao, Bülthoff, & Bülthoff, 2016b). However, the finding first and foremost shows that a good Gestalt-like fit of local shape, skin tone, and texture is seemingly not necessary for integrating upper and lower halves. It does not imply that Gestalt-like grouping is, in principle, not at play.

Curby and Entenman (2016) studied how the composite face effect depends on i) alignment/nonalignment of outer face frame and ii) alignment/nonalignment of the inner cardinal face features eyes, nose, and mouth. Results showed a strong decline of the composite face effect when the outer face frame was misaligned, while the cardinal face features remained aligned. Misaligning the cardinal face features while keeping the outer face frame aligned led to an even stronger reduction. Misaligning both did not diminish the congruency effect further. These findings show that the correct configural order of the cardinal face features is crucial for holistic integration, and also the connectedness of face outline is important. The findings also show that the factors behind holistic processing interact in a highly nonlinear fashion, since factors which show strong effects in isolation have weakened effect when other major drivers of holistic processing are already present. Our findings demonstrate that if cardinal facial features and face outline align, holistic integration is already robust and is not impaired by local breaks in nose shape and misfit in surface cues.

Studies on the inversion effect, which is another marker of configural sensitivity for faces (Maurer et al., 2002), yielded mixed findings concerning the role of facial feature shape and surface cues. Particularly the relevance of texture cues is less clear (Caharel, Jiang, Blanz, & Rossion, 2009; Hole, George, & Dunsmore, 1999; Russell, Biederman, Nederhouser, & Sinha, 2007). Russell, Biederman, Nederhouser, and Sinha (2007) found equally strong inversion effects for faces differing only in texture and faces differing only in shape. Comparing real face photographs and line drawings, Leder (1996) and Leder (1999) reported better detection of configural changes in real face images. Likewise, Meinhardt-Injac, Persike, and Meinhardt (2013) found inversion effects and facial feature summation which was modulated by face orientation only for real face images, while orientation dependent processing was absent for line drawings. However, results from other studies (Caharel et al., 2009; Jiang, Dricot, Blanz, Goebel, & Rossion, 2009) indicated that it might be 3D shape information contained in shading and surface texture, which is crucial for face-specific processing. This was substantiated by a recent study of Zhao, Bülthoff, and Bülthoff (2016b). Authors found that reducing real face images to 2D line drawings abolished the composite face effect, whereas removing all texture and color information while retaining a naked 3D head led to the same composite effects as real face images. These findings show that skin texture and shading per se are unimportant for holistic integration of face parts, but they can become important when they convey the 3D shape information necessary to recognize the complex stimulus as a human facial unity. The finding that crude face images with highly variable low-level image properties apparently tap face-specific routes also suggests that global face shape of a stimulus is one major factor for face-tuned processing (Tong, Nakayama, Moscovitch, Weinrib, & Kanwisher, 2000). These results indicate that global face shape of a stimulus and the correct configural order of its cardinal features essentially determine the perceptual integration of faces.

Zhao et al., (2016a) proposed that holistic processing is mediated both by bottom-up element grouping and top-down knowledge about which elements together constitute meaningful objects, and which perceptual strategies are appropriate in a given experimental task. There is ample evidence for a top-down route in face processing, since many studies have shown that the degree of holistic processing is modulated by cues to applying global or piecemeal strategies (Gao, Flevaris, Robertson, & Bentin, 2011; Meinhardt, Persike, & Meinhardt-Injac, 2014), by cues to grouping or segregating top and bottom parts (Curby, Goldstein, & Blacker, 2013), by target half certainty and feedback about correctness (Meinhardt et al., 2014; Meinhardt-Injac, Persike, & Meinhardt, 2014), by age-related loss of attentional control (Meinhardt-Injac et al., 2014; Meinhardt-Injac, Boutet, Persike, Meinhardt, & Imhof, 2017) and by the amount of learning experience with the stimulus material (Gauthier et al., 2003; Chua & Gauthier, 2019). Zhao and Bülthoff (2017) stressed that the interaction of factors driving holistic processing is highly nonlinear, which also concerns the interaction among top-down and bottom-up factors. Particularly, if holistic processing is already strong and not weakened by complex task constraints, adding additional cues does not necessarily augment the integration of face parts. As our results show, taking action to enhance fit quality of upper and lower halves in a classical composite face task with known target half does not lead to stronger holistic grouping than a random selection of face halves from the database. Methodologically, this finding could bring relief to researchers who rely on unconstrained selection of face halves when creating composite faces.

### Open Practices Statement

Data and materials for the experiment can be made available on author request. None of the experiments was preregistered.

## Footnotes

1 Considering the alignment effect only for “same” trials raises concerns from experimental methodology. In a forced-choice experiment with target and non-target trials, above-chance performance can only be ascertained by analyzing accuracy from both trial types. If accuracy from only one trial type is analyzed, it cannot be excluded that the observer is just guessing, reaching above chance performance for “same” at the costs of below chance performance for “different” trials (see MacMillan & Creelman, 2005, p. 6). Several authors who also used a reduced version of the design solved this problem by calculating accuracy from both “same” and “different” trials, and successfully reported the alignment effect as the measure for the composite face effect (see Discussion in Konar et al., 2013). For a comprehensive discussion of design issues for measuring the composite effect, see Meinhardt, Persike, and Meinhardt-Injac (2014) and Meinhardt, Persike, and Meinhardt-Injac (2017).

2 Several authors distinguish first and second presentation with different terms. Rossion and Boremanse (2008) called the first composite face “target face” and the second one “probe face”. We adopt the terminology of Richler, Tanaka, Brown, and Gauthier (2008) who called the first stimulus presentation “study” and the second one “test”, since these terms are meanwhile frequently in use.

3 To see this from Fig. 2 in Richler et al., (2008), verify that 1st and 2nd data column together reflect the alignment × congruency interaction for our Experiment 2, while 1st and 4th data column together reflect this interaction for our experiment I.
